# Genital inflammation screening for predicting sexually transmitted infections and bacterial vaginosis: an updated cost analysis from the GIFT study in Madagascar, South Africa, and Zimbabwe

**DOI:** 10.1186/s12905-026-04428-9

**Published:** 2026-05-14

**Authors:** Elise Smith, Aina Harimanana, Tinashe Mwaturura, Vaomalala Raharimanga, Theodora Mayouya Gamana, Katherine Gill, Emma Harding-Esch, Tania Crucitti, Lindi Masson, Jo-Ann Passmore, Edina Sinanovic

**Affiliations:** 1 Health Economics Unit, School of Public Health, Faculty of Health Sciences, University of Cape Town, Cape Town, South Africa; 2 Institut Pasteur de Madagascar, Antananarivo, Madagascar; 3 Organization for Public Health Interventions and Development, Harare, Zimbabwe; 4 The Biomedical Research and Training Institute, Harare, Zimbabwe; 5 Desmond Tutu HIV Centre, University of Cape Town, Cape Town, South Africa; 6 London School of Hygiene and Tropical Medicine, London, England; 7 Institute of Infectious Disease and Molecular Medicine (IDM), University of Cape Town, Cape Town, South Africa; 8 Women’s, Children’s and Adolescents’ Health and Disease Elimination Programs, Life Sciences Discipline, Burnet Institute, Melbourne, VIC Australia; 9 National Health Laboratory Service, Johannesburg, South Africa

**Keywords:** Sexually transmitted infections, Bacterial vaginosis, Screening, Genital inflammation, Point-of-care testing, Cost analysis, Women's health, Sub-Saharan Africa

## Abstract

**Background:**

Sexually transmitted infections (STIs) and bacterial vaginosis (BV) are significant public health issues, particularly in sub-Saharan Africa, and are associated with genital inflammation and increased HIV acquisition risk. A substantial proportion of these infections are asymptomatic, limiting the effectiveness of syndromic management. The Genital InFlammation Test (GIFT), a novel rapid point-of-care (POC) test, was developed to detect elevated inflammatory biomarkers associated with genital inflammation. The first-in-field prototype of the GIFT device was evaluated in a multicenter observational study conducted in South Africa, Zimbabwe, and Madagascar. This study updates prior cost estimates using a hypothetical GIFT prototype in South Africa and extends the analysis to routine family planning services in Madagascar, and Zimbabwe.

**Methods:**

A provider-perspective, combining a top-down and bottom-up costing approach, was conducted at device evaluation observational study sites in Madagascar, South Africa, and Zimbabwe (*n* = 1 per country). Economic costs, including capital and recurrent expenditures, were collected through facility records, interviews, and self-reported provider timesheets to determine the incremental cost of integrating GIFT screening into family planning consultations. Research-related costs were excluded. A probabilistic sensitivity analysis using Monte Carlo simulation was performed to address parameter uncertainty, particularly around GIFT’s estimated market price of US$5.00.

**Results:**

The incremental cost per woman screened with GIFT was estimated to be US $6.46 (95% CI: US $1.98 – US $12.22) in Madagascar, US $9.05 (95% CI: US $3.78 – US $15.83) in South Africa, and US $8.28 (95% CI: US $3.04 – US $16.52) in Zimbabwe, slightly higher than previous estimates for South Africa (US $3.53 - US$ 5.32). Recurrent costs (personnel, supplies, and overheads) constituted more than 98% of this cost, with the GIFT device being the primary cost driver.

**Conclusions:**

Updated costs suggest slightly higher implementation costs than previous estimates. This analysis suggests that the affordability and potential scale-up of GIFT and other novel POC screening tools will depend heavily on their final market price. These findings provide essential economic evidence to inform further analyses on cost-effectiveness, affordability, and optimal integration of GIFT into routine sexual and reproductive healthcare services in low- and middle-income countries.

**Supplementary Information:**

The online version contains supplementary material available at 10.1186/s12905-026-04428-9.

## Introduction

Sexually transmitted infections (STIs) remain a public health concern globally, with the most recent evidence from the World Health Organization (WHO) estimating that approximately one million new infections of the four most prevalent curable STIs, *Chlamydia trachomatis* (CT), *Neisseria gonorrhoeae* (NG), *Trichomonas vaginalis* (TV) and syphilis are acquired worldwide daily [1]. In women, untreated infections with CT, NG and TV can lead to serious complications such as pelvic inflammatory disease, infertility, and ectopic pregnancy and have been associated with preterm birth and low birth weight [2–4]. These STIs are also associated with a higher risk of acquiring and transmitting other STIs, including HIV, with untreated infections sustaining further transmission [5].

Bacterial vaginosis (BV) is a common, recurring vaginal condition, affecting approximately one in four women of reproductive age that involves a disruption of the normal vaginal microbiome which may cause symptoms such as abnormal discharge and odor while it remains asymptomatic in other cases [6]. The frequent recurrence of BV contributes substantially to psychological burden and diminished quality of life, while also increasing the likelihood of sequalae such as pelvic inflammatory disease, spontaneous abortion, and preterm birth as well as increased risk of STI acquisition, including HIV [7–12].

A substantial proportion of both STIs and BV present without symptoms, especially in women and girls. When symptoms do occur, they are often non-specific and may not be perceived as abnormal, complicating case detection and clinical management [13]. A recent meta-analysis estimated that 61% of CT, 53% of NG and 57% of TV infections in women from LMICs are asymptomatic, with even higher proportions among women in Africa (69%, 67%, and 65%, respectively) [14]. Estimates for asymptomatic BV range from 47% to 50% in LMICs, while studies in high income settings have found it to be above 80% [15–18].

WHO has set ambitious targets aimed at reducing the global incidence of STIs by 2030, emphasizing the need to scale-up prevention and control measures, particularly in sub-Saharan Africa, which bears the highest burden worldwide [1]. Progress towards these goals, however, remains challenging, in part due to limited diagnostic capacity in many low- and middle-income countries (LMICs). In addition, these targets do not encompass BV, despite its substantial global prevalence and comparable adverse effects on reproductive health outcomes to those of STIs [6]. In settings where syndromic management remains the standard of care its limitations are significant: it has low predictive value for discharge-causing STIs such as CT, NG, TV and for BV and it is unable to identify asymptomatic infections [13].

Both STIs and BV are known to induce genital inflammation in some cases, regardless of symptoms [4, 19]. Genital inflammation, in turn, is strongly associated with an increased risk of HIV acquisition (up to 3.2-fold) and adverse birth outcomes, such as preterm birth [10, 20]. This is particularly relevant in sub-Saharan Africa, where women are disproportionately affected by curable STIs and a substantial proportion of new HIV infections occur in women [13, 21]. However, progress in developing point-of-care (POC) rapid diagnostics for CT, NG, TV and BV that fulfil WHO’s REASSURED criteria (including being affordable, rapid, equipment-free and deliverable to end-users) has been limited, leaving a critical gap in effective disease management [22, 23]. Affordable diagnostic tools capable of delivering same-day results remain a pressing research and program priority, as they are essential for enabling timely initiation of appropriate treatment and reducing onward transmission [24, 25]. To address this need, Masson and colleagues developed the Genital InFlammation Test (GIFT). GIFT is a rapid POC lateral flow assay detecting inflammatory biomarkers interleukin (IL)-1α, IL-1β, and interferon-γ-induced protein (IP)-10 from vaginal swabs and producing results in 15 min [21]. A positive GIFT test would require follow-up testing laboratory-based or near–point-of-care diagnostics (e.g. GeneXpert) to identify the specific STIs and guide appropriate treatment. By screening for genital inflammation, GIFT could facilitate the identification of women with asymptomatic STIs and BV and at elevated risk of HIV acquisition. This approach has the potential to strengthen sexual and reproductive health outcomes in settings where syndromic management is still the standard of care through identifying asymptomatic infections and reducing the number of individuals requiring more costly diagnostic testing. The GIFT study, a multicenter observational study conducted in South Africa, Zimbabwe, and Madagascar, evaluated the first-in-field prototype of the GIFT device. Its primary objective was to assess the sensitivity and specificity of GIFT for detecting asymptomatic, discharge-causing STIs (CT, NG, TV, *Mycoplasma genitalium* (MG)) and BV in women aged 18–35 years accessing family planning services, using nucleic acid amplification tests (NAATs) and Nugent scoring as reference standards [21].

A key objective of the larger GIFT study was to assess potential pathways for integration into family planning services, including an analysis of what implementing GIFT screening in real-world clinical settings might cost. An earlier study estimated the costs of GIFT screening for STIs and BV during family planning consultations in South Africa, based on a hypothetical prototype of the GIFT device that was still under development [26]. Cost estimates were derived from three health facilities in Cape Town, including a government clinic, a non-governmental clinic, and a semi-private clinic. The incremental cost of incorporating GIFT screening for the detection of asymptomatic STIs and BV during family planning consultations was estimated between US $3.53 (government clinic) and US $5.32 (semi-private clinic) (2016 prices inflated to 2023). This included a device price of US $0.30 based on manufacturer’s price estimates of the GIFT [26].

The primary aim of this study was to provide an updated estimate of the incremental cost of GIFT screening (testing of individuals who do not show symptoms to identify infections early and prevent transmission and complications) for STIs and BV in a real-world implementation setting, including South Africa and extending this analysis to Madagascar and Zimbabwe, based on evidence from the multicenter clinical observational GIFT device accuracy study [21].

## Methods

### Study design

A combined top-down and bottom-up costing approach was applied to estimate the incremental cost per woman, and per woman per year, screened for STIs and BV using GIFT as part of routine family planning services in the three study settings. Economic costs, including both capital and recurrent costs, were assessed from the provider perspective. All research-related study costs were excluded so that resource utilization reflects the most likely scenario of routine implementation of GIFT screening in a real-world setting.

### Study sites

The GIFT study was conducted at three sites across the three countries (one per country). In Madagascar, the study site was the Centre Hospitalier Universitaire de Gynécologie et d’Obstétrique de Befelatanana, a government university hospital in Antananarivo where family planning services are provided in the outpatient department of the hospital alongside other SRH and maternal health services. The Desmond Tutu Health Foundation (DTHF) Masiphumelele Youth Centre, a non-profit, donor-funded research center in Cape Town, was South Africa’s study site. DTHF operates an on-site clinic where youth-friendly sexual and reproductive health (SRH) services, including family planning and STI management, are provided. Given that DTHF is a donor-funded clinic, cost and service utilization data on family planning services were obtained from the nearby government Masiphumelele clinic, a public health clinic offering integrated family planning and STI services, to estimate costs that are more representative of a public health setting. All data related to the GIFT study were obtained from the DTHF GIFT study site. In Zimbabwe, the study was conducted at the Spillhaus Family planning Centre, a public clinic in Harare offering integrated SRH services including family planning, STI management, HIV prevention and fertility services. Across all three countries and study sites, syndromic management of STIs and BV remains the standard of care.

### Data collection

Cost and resource utilization data of family planning consultations were obtained from facility management at each of the health facilities using semi-structured interview tools. Time allocation of clinical personnel to family planning services was collected through self-reported timesheets over a two-week period per site. Similarly, information on the time and consumables required to conduct GIFT screening was collected using self-reported timesheets completed by GIFT study nurses at each study site. For the base-case analysis, the GIFT device was assigned a cost of US $5.00, reflecting the manufacturer’s current best estimate of a potential market price and in line with the WHO’s guidance on the target price for CT and NG POC tests in LMIC and public sector contexts.

Where data were unavailable from the study sites, publicly accessible data sources or comparable data from similar study contexts were utilized (Table 1).

**Table 1:** Measurement, valuation, and allocation of costs to GIFT screening

Cost category	Measurement		Valuation		Allocation to GIFT screening	
	Method	Data source	Method	Data source	Method	Allocation factor
Capital cost items						
Buildings	Square meters of space used for FP consultations	Obtained from facilities	Current replacement value (cost per m^2^)	[27]*	Top-down	FP space as % of total facility space x *GIFT screening personnel time as % of total direct personnel time for FP*
			Useful life (20 years)	[28]		
			Discount rate (3%)	[29, 30]		
Equipment (medical & non-medical) and furniture	Items used for FP departments	Obtained from facilities**	Current replacement value	Health facility; market prices	Top-down	Total FP consultations as % of total outpatient consultations x *GIFT screening as % of total direct personnel time for FP*
			Useful life (15 years for furniture, 5 years for equipment)	[28]		
			Discount rate (3%)	[29, 30]		
Provider training	Provider time	GIFT clinical study team	Total annual renumeration	Government salary scales	Bottom-up	Fully allocated to GIFT screening
			Useful life (5 years)	[28]		
			Discount rate (3%)	[29, 30]		
*Recurrent cost items*						
Indirect personnel	Time spent on FP- related support activities	Self-reported timesheets	Total annual renumeration	Government salary scales	Top-down	Total FP consultations as % of total outpatient consultations x *GIFT screening as % of total direct personnel time for FP*
Direct personnel	Time spent on GIFT screening per study participant^¥^	Self-reported timesheets	Total annual renumeration	Government salary scales	Bottom-up	Direct; based on reported utilization
Medical and non-medical supplies	Supplies used for conducting a GIFT test	Self-reported reports	Unit prices	Health facility; market prices	Bottom-up	Direct; based on reported utilization
Overheads & maintenance	Apportioned usage	Obtained from facilities**	Total annual expenditure	Facilities	Top-down	Total FP consultations as % of total outpatient consultations x *GIFT screening as % of total direct personnel time for FP*

*FP* family planning, *GIFT *Genital InFlammation Test

*Source for the replacement value of the clinic in South Africa. In the absence of data for Madagascar and Zimbabwe, building costs were estimated by applying the proportion of building costs of total unit costs as in South Africa

** In the absence of available data from the clinic in South Africa, information was sourced from Kairu et al. [26] and adjusted to 2023 prices

^¥^ Excludes study-related activities such as explaining study procedures, obtaining consent, and collecting additional swabs

### Data analysis

Data were analyzed using a costing model developed in Microsoft Excel 365. The total cost of family planning and incremental cost of GIFT screening were estimated at the facility level. The incremental cost of GIFT screening was divided by the total number of female patients attending the clinic in the 2023 fiscal year to estimate the incremental cost of GIFT screening per woman. The total cost per woman screened per year was calculated as the product of the incremental cost per woman screened and the number of family planning consultations per woman per year.

All primary costs and utilization data was collected for the 2023 financial year, and costs are subsequently presented in 2023 United States Dollars (US $). Costs were inflated to 2023 prices using locally published inflation data where required and converted to US $ using the published average annual exchange rate for each country for the period 1 January 2023 to 31 December 2023 (US $1 = Malagasy Ariary 4,454.62, US $1 = South African Rand 18.46, US $1 = Zimbabwean Dollar 3,680.52 [31–33].

### Measurement, valuation, and allocation of costs

Capital cost items included buildings, equipment, furniture, and provider training, while recurrent cost items comprised personnel, supplies, and overheads. Table 1 outlines the approach used to measure, value, and allocate these costs to GIFT screening consultations.

Capital cost utilization was measured using facility records and interviews with the GIFT study team, and valued using current replacement costs from facility data, market prices, or published sources. These costs were annualized using commonly accepted assumptions on useful life and a 3% discount rate (see Table 1) [29]. Recurrent cost utilization was primarily measured through self-reported timesheets and facility records. These costs were valued using government salary scales, market prices, and facility expenditure data.

Indirect and shared resources were allocated to the GIFT screening consultation using top-down approaches: Building costs were allocated according to the proportion of space used for family planning consultations, further apportioned to GIFT screening based on the share of direct personnel time devoted to GIFT as a proportion of total family planning consultation time; Equipment, furniture, indirect personnel, and overhead and maintenance costs were allocated in proportion to family planning consultations as a share of total outpatient visits. In Madagascar, hospital-wide shared costs (including overheads and maintenance) were allocated to family planning consultations based on the proportion of these visits relative to the total hospital service volume. To ensure comparability between service types, inpatient days were converted to outpatient-equivalent visits using WHO-CHOICE conversion factors [34]. Direct resources, including GIFT provider training, direct personnel time, and medical and non-medical supplies, were allocated directly to GIFT screening. 

### Sensitivity analysis

Univariate sensitivity analysis was conducted to identify the most influential parameters on the results of the analysis. Model parameters were varied to ± 30% of the base-case scenario based on expert opinion. Results from this analysis are presented by means of tornado plots.

### Uncertainty analysis

Given the small sample of this study, significant uncertainty existed around the base-case point estimates and the generalizability thereof to other settings within the same country. Parameter uncertainty was characterized by using probabilistic sensitivity analysis (PSA) via Monte Carlo simulation with 1,000 iterations. Gamma or uniform distributions were assigned to cost input parameters, while lognormal or uniform distributions were applied to resource utilization parameters [35]. The limited sample size in this study, both in terms of the number of facilities and the number of personnel from whom time estimates were collected, precluded the statistical calculation of means and 95% confidence intervals (CIs) for cost model parameters based on observed data. Consequently, base-case cost parameters primarily reflect single-point estimates or simple averages derived from the available observations (e.g., time measurements) or data collected from the facilities. Where possible, upper, and lower empirical bounds for model parameters were derived from minimum and maximum values based on observed data (provider time spent family planning and time taken to perform a GIFT test) or proxies based on published guidance or literature (GIFT device price and frequency of consultations), The GIFT test price was ranged from US$1 to US$10 to reflect (i) the WHO “optimal” affordability target at the lower bound [36] and (ii) a conservative upper bound that allows for pre-market uncertainty while remaining within a plausible public-sector affordability envelope based on expert opinion. The cadence of common contraceptive method (e.g. bi-monthly injections, oral contraceptives supply received six-monthly) informed uncertainty ranges presenting likely minimum and maximum number of consultations, and to allow for the fact that some women might not attend appointments regularly, while others might seek care more frequently. Thus, varying the number GIFT screenings women would likely receive in a year under the assumption of universal screening. In the absence of empirical bounds, a ± 30% variation around the base-case estimate of other model parameters was assumed to parameterize the PSA model, in alignment with the ranges applied in the one-way sensitivity analysis [37]. Results from the PSA are presented as 95% CIs around the base-case point estimates.

### Ethical considerations

Ethical approval for this study was granted by the following research ethics committees: University of Cape Town (UCT) (Ref: 366/2022 and 173/2023), London School of Hygiene & Tropical Medicine (Ref: 28046), Ministère de la Santé Publique (Ref: 64-MSANP/SG/AMM/CNPN/CERMB), City of Cape Town (Ref: 9870) and, Research Council of Zimbabwe (Ref: 04768). The study was conducted in accordance with the ethical principles outlined in the Declaration of Helsinki, and all applicable national and institutional guidelines for research involving human participants were followed.

### Consent to participate

All human participants provided informed consent to participate in this research as part of the GIFT multicenter observational study (EDCTP-RIA2020I-3297).

## Results

Facility and resource utilization, which informed the allocation of shared resources to family planning and ultimately GIFT screening, is presented in Table 2. The proportion of family planning consultations relative to total outpatient visits varied substantially across sites, accounting for 16% in Madagascar, 12% in South Africa, and 88% in Zimbabwe. Across the three facilities, clinical personnel (nurses) spent 71%, 84% and 78% of their time on activities related to family planning services, respectively. This includes time spent on clinical (client-facing) and non-clinical activities (such as case management and administrative tasks) attributed to family planning services as well as idle time.

**Table 2:** Facility and resource utilization

	Madagascar		South Africa			Zimbabwe		
	Point estimate (min - max)	*n*	Point estimate (min - max)		*n*	Point estimate (min - max)	*n*	Source
Family planning (FP)								
Total FP consultations, per year	4 135 (2 895–5 376)	1	4 727 (3 309–6 145)		1	2 263 (1 584–2 942)	1	Facility records*
Total number of outpatient visits per year	25 229 (17 660–32 798)	1	40 907 (28 635–53 179)		1	2 586 (1 810–3 362)	1	Facility records*
Total number of inpatient days per year	56 565 (39 596–73 535)	1	n/a		1	n/a	1	Facility records*
FP consultations as % of outpatient visits	16%	1	12%		1	88%	1	Calculated from above values
% of total direct personnel time spent on FP (of all routine tasks, excluding GIFT screening)	71% (65% − 77%)	2	84% (71% − 98%)		4	78% (71% − 81%)	4	Provider timesheets^¥^
Total FP consultations (and GIFT screenings), per woman, per year	4 (2–6)	1	4 (2–6)		1	4 (2–6)	1	Model assumption**
GIFT screening								
Average time per GIFT test performed (minutes)^α^	11.67(9.00–17.00)		(*n* = 6)					Provider timesheets^¥^

*n/a* Not applicable

*Probabilistic sensitivity analysis minimum and maximum values represent +/-30% of reported parameters

^**^ Probabilistic sensitivity analysis minimum and maximum values are based on the cadence of common contraceptive method, presenting likely minimum and maximum number of consultations, and thus GIFT screenings, per year per type of contraceptive method

^α^ Due to the small number of observations across study sites, the average time to perform a GIFT test represents the average observations from across the three study sites. This estimate excludes the incubation time of the GIFT device based on the assumption that clinical personnel would be able to carry out other routine family planning consultation tasks while waiting for test results

^¥^ Probabilistic sensitivity analysis minimum and maximum values from data collected

In the base-case analysis, each woman was assumed to attend four family planning consultations per year, reflecting an average utilization pattern of one clinic visit every three months. Women were assumed to be screened for genital inflammation with the GIFT test at each family planning visit, with this assumption tested in sensitivity and uncertainty analyses.

The average time taken by nurses to perform a GIFT test (including explaining the test to the client, collecting the vaginal swab, mixing with buffer and applying it to the test device and reading and interpreting results) excluding research-specific activities and the 15-minute test incubation period, was 11.67 min across the three sites. This is slightly higher than the 10 min estimated by Kairu et al. [26]. Due to the small number of observations across study sites, the average time across observations from all three study sites was applied to the cost estimates for all sites. Incubation time of the GIFT device was excluded based on the assumption that clinical personnel would be able to carry out other routine family planning consultation tasks while waiting for test results. 

Table 3 presents the base-case unit cost estimates and the 95% CIs from the PSA. The incremental cost per woman screened with GIFT was estimated to be US $6.46 (US $1.98 – US $12.22) in Madagascar, US $9.05 (US $3.78 – US $15.83) in South Africa and US $8.28 (US $3.04 – US $16.52) Zimbabwe. Recurrent costs accounted for more than 98% of total unit costs in all three countries, with capital costs comprising the remainder. The GIFT device is the primary driver of the incremental cost of GIFT screening at an estimated unit price of US $5.00 (77% in Madagascar, 55% in South Africa and 60% in Zimbabwe). To reflect current uncertainty, a price range between US $1.00 and US $10.00 was applied for the device (refer to *Sensitivity Analysis*). The second biggest cost driver was medical supplies in Madagascar (15.3%), direct personnel in South Africa (29.5%), and indirect personnel in Zimbabwe (19.0%).

**Table 3:** Unit cost results (2023 US $)

	Madagascar	South Africa	Zimbabwe	Madagascar	South Africa	Zimbabwe	Madagascar	South Africa	Zimbabwe
	Incremental cost per woman screened with GIFT, US $ (95% CI)			Incremental cost per woman screened with GIFT, per year, US $ (95% CI)			Cost component as % cost per woman screened (and cost per year)		
Capital costs									
Buildings	0.07 (0.02–0.29)	0.10 (0.05–0.28)	0.09 (0.03–0.39)	0.29 (0.08–0.90)	0.41 (0.12–1.32)	0.38 (0.10–1.69)	1.1%	1.1%	1.1%
Equipment and furniture	0.003 (0.001–0.007)	0.001 (0.0004–0.002)	0.05 (0.02–0.12)	0.01 (0.007–0.02)	0.003 (0.001–0.01)	0.19 (0.12–0.28)	0.05%	0.01%	0.06%
Procedure training	0.001 (0.0007–0.002)	0.01 (0.01–0.02)	0.01 (0.004–0.01)	0.004 (0.002–0.01)	0.05 (0.02–0.10)	0.03 (0.01–0.05)	0.02%	0.1%	0.1%
*Total capital costs*	*0.* *08* *(0.02–0.29)*	*0.12* *(0.06–0.30)*	*0.15* *(0.06–0.52)*	*0.31* *(0.09–0.93)*	*0.47* *(0.13–1.42)*	*0.59* *(0.23–2.02)*	*1.2%*	*1.3%*	*1.8%*
*Recurrent costs*									
Indirect personnel	0.08 (0.04–0.19)	0.26 (0.14–0.60)	1.57 (0.87–3.80)	0.31 (0.23–0.42)	1.04 (0.34–3.04)	6.24 (4.70–8.66)	1.2%	2.9%	19.0%
Direct personnel	0.32 (0.22–0.44)	2.67 (1.87–3.55)	0.49 (0.12–0.68)	1.26 (0.51–2.30)	10.68 (4.45–18.90)	1.94 (0.81–3.65)	5.0%	29.5%	5.9%
Medical supplies	0.99 (0.70–1.30)	0.99 (0.71–1.33)	0.99 (0.71–1.29)	3.95 (1.60– 7.10)	3.95 (1.64–7.16)	3.95 (1.68–7.15)	15.3%	10.9%	12.0%
GIFT device	5.00 (1.00–10.00)	5.00 (1.00–10.00)	5.00 (1.00–10.00)	20.00 (3.00–54.00)	20.00 (3.00–54.00)	20.00 (2.00–54.00)	77.4%	55.2%	60.4%
Overheads and maintenance	0.0005 (0.0002–0.001)	0.02 (0.01–0.04)	0.09 (0.04–0.24)	0.002 (0.001–0.003)	0.07 (0.02–0.22)	0.38 (0.24–0.57)	0.01%	0.2%	1.1%
*Total recurrent costs*	*6.38* *(1.96–11.92)*	*8.93* *(3.72–15.53)*	*8.13* *(2.98–16.00)*	*25.52* *(5.33–63.82)*	*35.73* *(9.46–83.31)*	*32.54* *(9.44–74.03)*	*98.8%*	*98.7%*	*98.2%*
*Total costs*	*6.4* *6* *(1.98–12.22)*	*9.05* *(3.78–15.83)*	*8.28* *(3.04–16.52)*	*25.83* *(5.42–64.75)*	*36.20* *(9.60–84.73)*	*33.13* *(9.67–74.03)*	*100%*	*100%*	*100%*
*Total cost*,* excluding GIFT device*	*1.46* *(0.98–2.22)*	*4.05* *(2.78–5.83)*	*3.28* *(2.04–6.52)*	*5.83* *(2.83–10.75)*	*16.20* *(6.60–30.73)*	*13.13* *(6.60–20.03)*	*22.6%*	*44.8%*	*39.6%*

Differences in cost estimates between the three countries were driven by variations in price levels, particularly those related to human resources, as well as differences in the allocation factors of shared costs to family planning and, ultimately, to GIFT. In Zimbabwe, where 88% of facility costs were allocated to family planning the indirect staff costs attributed to GIFT were substantially higher than in the other countries. The higher family planning utilization factor observed in Zimbabwe reflects the Spillhaus facility’s primary focus on family planning, with limited provision of other SRH services. This affects the allocation of equipment, overhead, maintenance, and indirect personnel costs to GIFT screening, with equipment costs being the only component that substantially influences estimated unit costs (Tables 2 and 3). In facilities where a smaller share of total visits is attributable to family planning, similar to Madagascar and South Africa, unit costs in Zimbabwe would be expected to decline to levels comparable with Madagascar. In South Africa, where human resource costs were higher than in Madagascar and Zimbabwe, direct personnel costs were greater despite the same level of direct resource utilization being assumed for GIFT screening due to salary differentials. To facilitate further comparison of costs, these are also presented in 2023 international dollars in Supplementary Table 1.

The incremental cost per woman screened per year, assuming routine screening at each of four family planning visits, was estimated to be US $25.83 (US $5.42 – US $64.75) in Madagascar, US $36.20 (US $9.60 – US $84.73) in South Africa and US $33.13 (US $9.67 – US $74.03) in Zimbabwe.

### Sensitivity analysis

**Fig. 1:**
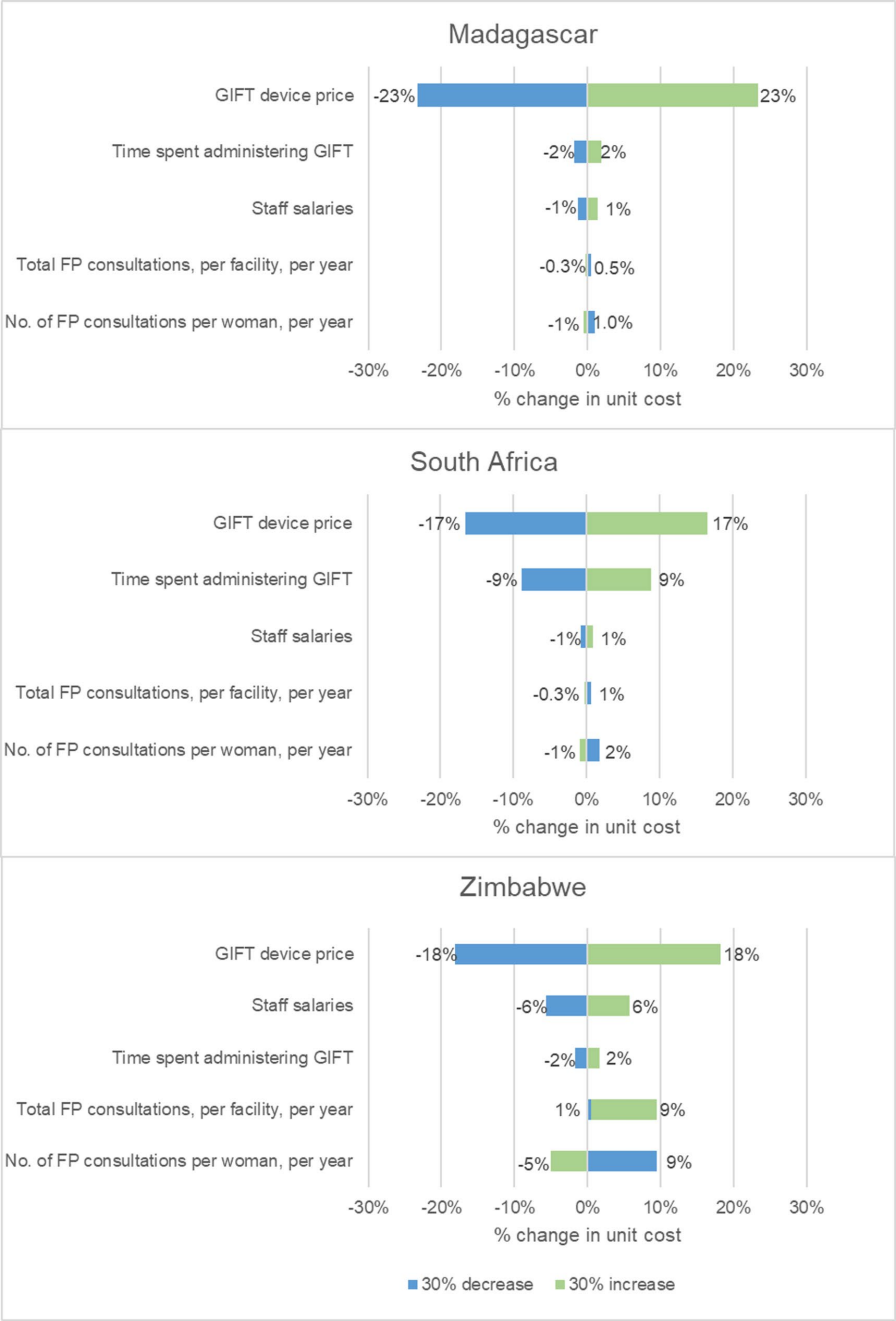
Univariate Sensitivity Analysis. Note: FP = family planning GIFT = Genital InFlammation Test; Across countries, the GIFT device price had the greatest impact on unit cost; a ±30% change led to ±23%, ±17%, and ±16% changes in incremental cost per woman screened in Madagascar, South Africa, and Zimbabwe, respectively. The next most influential parameters were administration time (Madagascar, South Africa) and the number of FP visits per woman per year (Zimbabwe)

Figure 1 shows the results from the univariate sensitivity analysis where model parameters were varied with ± 30%. Across the three countries, a change in the GIFT device price resulted in the greatest change in unit cost, with a ± 30% change resulting in a ± 23%, ± 17% and ± 16% change in the incremental cost per woman screened in Madagascar, South Africa and Zimbabwe, respectively. The second most influential parameter was the time required to administer the GIFT device in Madagascar and South Africa, and the number of family planning consultants per woman, per year in Zimbabwe. The inverse relationship between the number of family planning consultations per woman per year and the cost of GIFT screening suggests that economies of scale could be realized by distributing fixed costs across a larger number of services, albeit relatively modest.

## Discussion

This study provides a multi-country cost analysis of implementing GIFT, a rapid POC test for genital inflammation, or other similar tests, as an STI screening tool within routine family planning services in Madagascar, South Africa, and Zimbabwe. The incorporation of PSA allows for the quantification of uncertainty around point estimates, reflecting the inherent variability in cost parameters, especially given the small sample size of health facilities.

In our base case analysis, the cost per women screened was estimated at US $6.46 in Madagascar, US $9.05 in South Africa and US $8.28 in Zimbabwe (US $1.46, US $4.05 and US $3.28, respectively, excluding the cost of the GIFT device). These estimates are slightly higher than the inflation-adjusted results reported by Kairu et al. based on the hypothetical implementation of GIFT South African government clinic: US $3.53, or US $3.23 excluding the device price of US $0.30 ) [26]. The observed differences in cost excluding the device are primarily attributable to variations in human resource utilization between the two studies. Overall cost estimates, including the device, were higher in our analysis largely due to the increased device price assumed, based on more recent manufacturer estimates. Using empirical cost data from three LMICs enhances the relevance of our findings for similar contexts. However, data were collected from a single urban facility per country, limiting generalizability. Urban facilities typically serve higher patient volumes than rural or peri-urban sites, where lower volumes may increase unit costs through diseconomies of scale. Rural settings may also face higher personnel costs (e.g., South Africa where rural allowances are paid to healthcare workers [38]) and additional logistics and supply chain expenses, which were not accounted for in this analysis. As the main cost drivers were the GIFT device, medical supplies, and personnel time, variation in salaries and supply chain costs could meaningfully affect unit costs in rural and peri-urban settings. While the precise magnitude cannot be quantified with the available data, sensitivity analyses provide an indication of plausible cost ranges, with the upper bounds likely reflecting costs in more rural settings. Future implementation research should therefore include a broader range of facility types, including rural and lower-volume settings, to better characterise the full likely range of costs and to inform planning for scale-up across heterogeneous health system contexts.

The wide range of unit costs obtained in the PSA indicate reflect considerable uncertainty, largely attributable to the limited number of facilities sampled and uncertainty regarding the future public sector market price of the GIFT device. As the device cost is the main driver of total implementation costs in our analysis, the overall affordability and scalability of GIFT screening will depend largely on its eventual market price; a critical consideration for implementation in LMIC settings. In the base case analysis, the device price was assumed to be US $5.00. However, the final market price will ultimately depend on the device’s components and where they are manufactured.

Evidence on the costs of STI screening using POC tests in LMICs, including in Sub-Saharan Africa, remains limited and largely focused on HIV and syphilis. This is likely due to the lack of affordable and effective POC diagnostic tests for other STIs, including CT, TV and NG, and prevailing guidance to use syndromic management in these settings. To our knowledge, no published data currently exists on the cost of CT, NG, or TV screening using rapid POC tests in Africa or other LMICs. For context, a systematic review estimated the mean cost of facility-based HIV testing using rapid POC tests in Sub-Saharan Africa at US$19.63 per person tested (including post-test counselling), although self-testing approaches have achieved lower costs (US$12.75 per person tested) [39]. In LMICs, facility-based antenatal syphilis screening using rapid POC tests has been estimated at approximately US$1.00–5.00 per woman screened, with staff time and supplies, including the device itself, typically costing less than US$1.00, constituting the main cost drivers. When considering scale-up, screening or testing volumes are an important consideration, as higher volumes allow for economies of scale by spreading fixed costs such as training, start-up activities, facility operations, program supervision, and quality assurance, over a larger number of tests [40–50].

Overall, these findings suggest that GIFT screening compares favorably with facility-based HIV testing and self-testing, though generally higher than antenatal syphilis screening. If device costs can be reduced to the lower end assumptions and economies of scale achieved through scaled implementation, GIFT screening could represent an economically viable and feasible STI screening strategy in LMICs, with the potential to improve early detection and sexual and reproductive health outcomes. For example, the time per GIFT screening may decrease over time as initial learning curves associated with a new screening protocol are overcome and providers become more proficient in using the technology.

By varying the number of GIFT screening per woman per year in the sensitivity analysis we have considered how total annual costs would increase or decrease under different utilization scenarios, but further research is warranted to fully assess the total cost of implementing a universal screening program over time. For example, women might not attend family planning visits as frequent as four times a year, STI risk and healthcare seeking behavior might differ across settings, and desired screening frequency might change over time due to changes in STI prevalence in a population. Importantly, GIFT screening costs cannot be considered in isolation, as confirmatory diagnostic testing is required to guide appropriate treatment. Accurately estimating the full cost of implementation will therefore require a more detailed understanding of how GIFT could be integrated into routine SRH services. Finally, these cost considerations must be interpreted alongside forthcoming evidence of the device’s effectiveness and subsequent impact on health outcomes.

### Limitations

This study has several limitations. Data were collected from a single facility per country, which may not be representative of all public health facilities, limiting generalizability. Time allocation data relied on self-reported timesheets that were collected over a two-week period. Self-reporting may be subject to recall or reporting bias. In addition, the relatively short data-collection period for personnel time may not fully capture variation in workload over time. Furthermore, the US $5.00 price of the GIFT device is a manufacturer’s estimate, and the final market price could differ, substantially impacting overall and relative costs.

Where facility-specific data were unavailable, costs were imputed using published sources or proportional estimates from other sites, such as building costs in Madagascar and Zimbabwe, which may not fully reflect local conditions. Furthermore, primary cost data were collected in 2024, with 2023 representing the most recent year for which complete and validated expenditure, price, and utilization data were consistently available across all sites. Updating costs to a later year was not undertaken due to challenges in applying inflation and exchange rate adjustments, in Zimbabwe, which transitioned from the Zimbabwean dollar to the Zimbabwe Gold in April 2024. Presenting costs in 2023 USD was therefore considered the most methodologically appropriate approach to maintain internal consistency and comparability across the three country contexts. However, cost should thus be interpreted with caution, considering the inflationary and volatile foreign exchange markets in the countries included in the analysis.

Finally, this analysis did not include the costs of confirmatory testing for STIs and BV, which would form a key component of the management of STIs, and BV following positive GIFT test results and should be incorporated adequately in subsequent cost-effectiveness and budget impact analyses to inform any policy decisions around implementation of GIFT screening.

## Conclusion

This cost analysis demonstrates that incorporating GIFT screening into routine family planning services costs between US $6.46 and US $9.05 per woman screened across settings in Madagascar, South Africa, and Zimbabwe. This represents a higher estimate that previously estimated for South Africa (US $3.53 - US$ 5.32) explained mainly by higher assumptions around the price of the GIFT device. The cost of the device itself is the predominant cost driver, underscoring that the affordability of this novel POC technology will be central to its potential for widespread adoption. These findings provide essential evidence for the broader economic evaluation of implementing GIFT as a screening tool for STIs and BV in LMICs.

Improved detection and treatment of STIs through the detection of genital inflammation, can significantly reduce downstream costs of the treatment of sequalae of STIs and HIV infection acquired due to increased risk caused by STIs. While this study provides a quantitative assessment of the economic costs associated with GIFT implementation, further research is required to integrate these cost data with evidence on clinical effectiveness from the ongoing GIFT study as well as the total cost of implementing confirmatory diagnostic testing. Emerging results on cost-effectiveness, affordability, and acceptability will be essential to inform policy recommendations and guide the design of implementation strategies, such as optimizing screening frequency or targeting high-risk sub-populations to maximize health benefits within existing resource constraints. 

## Supplementary Information

Supplementary Material 1.

## Data Availability

Data and analysis generated in this study is available upon reasonable request from the corresponding author.

## Abbreviations

BV: Bacterial vaginosis
CT: Chlamydia trachomatis
DTHF: Desmond Tutu Health Foundation
FP: Family planning
GIFT: Genital InFlammation Test
HIV: Human immunodeficiency virus
IL-1α: Interleukin-1α
IL-1β: Interleukin-1β
IP-10: Interferon-γ-induced protein 10
LMICs: Low- and middle-income countries
MG: Mycoplasma genitalium
MGA: Malagasy Ariary
NAATs: Nucleic acid amplification tests
NG: Neisseria gonorrhoeae
POC: Point-of-care
PSA: Probabilistic sensitivity analysis
SRH: Sexual and reproductive health
STIs: Sexually transmitted infections
TV: Trichomonas vaginalis
US $: United States Dollar
WHO: World Health Organization
