# Supplementary material for: Genital inflammation screening for predicting sexually transmitted infections and bacterial vaginosis: an updated cost analysis from the GIFT study in Madagascar, South Africa, and Zimbabwe
Source: BMC Womens Health. 2026 May 14;26:340. doi: 10.1186/s12905-026-04428-9 (PMC13343555; doi:10.1186/s12905-026-04428-9)
Supplement: Supplementary file 1 — Supplementary Material 1. [file 12905_2026_4428_MOESM1_ESM.docx]

**Supplementary materials**

***Cost analysis of genital inflammation screening as a predictor of sexually transmitted infections and bacterial vaginosis in women: evidence from the GIFT study in Madagascar, South Africa, and Zimbabwe***

Unit cost results are converted to 2023 International Dollar values (Int $) based on PPP conversion rates published by the International Monetary Fund (Int $1 = Malagasy Ariary 1,250, Int $1 = South African Rand 7.01, Int $1 = Zimbabwean Dollar 2,520) (1). GIFT test costs are displayed in USD values.

Table 1: Unit cost results (2023 Int $))

|  | **Madagascar** | **South Africa** | **Zimbabwe** | **Madagascar** | **South Africa** | **Zimbabwe** |
| --- | --- | --- | --- | --- | --- | --- |
|  | **Incremental cost per woman screened with GIFT, Int $ (95% CI)** | | | **Incremental cost per woman screened with GIFT, per year, Int $ (95% CI)** | | |
| ***Capital costs*** | | | | | | |
| Buildings | 0.26 (0.07 – 1.02) | 0.27 (0.13 -0.74) | 0.14 (0.04 – 0.57) | 1.05 (0.13 – 6.11) | 1.08 (0.26 -4.46) | 0.55 (0.08 – 3.40) |
| Equipment and furniture | 0.01 (0.005 – 0.02) | 0.002 (0.002 – 0.01) | 0.07 (0.04 – 0.17) | 0.04 (0.01 – 00.14) | 0.01 (0.004 – 0.03) | 0.28 (0.07 – 1.03) |
| Procedure training | 0.004 (0.002 – 0.01) | 0.03 (0.03 – 0.05) | 0.01 (0.01 – 0.01) | 0.01 (0.005 – 0.0.03) | 0.13 (0.07 – 0.30) | 0.04 (0.01 – 0.09) |
| ***Total capital costs*** | ***0.27 (0.07 -*** | ***0.31 (0.16 – 0.80)*** | ***0.22 (0.08 – 0.75)*** | ***1.10 (0.15 – 6.28)*** | ***1.23 (0.33 – 4.79)*** | ***0.86 (0.17 – 4.52)*** |
| ***Recurrent costs*** | | | | | | |
| Indirect personnel | 0.27 (0.15 – 0.66) | 0.68 (0.37 – 1.58) | 2.29 (1.27 – 5.55) | 1.10 (0.30 – 3.96) | 2.73 (0.74 – 9.50) | 9.16 (2.54 – 33.28) |
| Direct personnel | 1.12 (0.80 – 1.57) | 7.03 (4.91 – 9.36) | 0.71 (0.52 – 0.99) | 4.50 (1.59 – 9.41) | 28.12 (9.83 – 56.14) | 2.84 (1.03 – 5.95) |
| Medical supplies | 3.52 (2.48 – 4.62) | 2.60 (1.86 – 3.51) | 1.44 (1.04 – 1.88) | 14.06 (4.97 – 27.71) | 10.39 (3.71 – 21.08) | 5.76 (2.08 – 11.27) |
| GIFT device | 5.00 (1.00 – 10.00) | 5.00 (1.00 – 10.00) | 5.00 (1.00 – 10.00) | 20.00 (2.00 – 60.00) | 20.00 (2.00-60.00) | 20.00 (2.00 – 60.00) |
| Overheads and maintenance | 0.002 (0.001 – 0.005) | 0.05 (0.02 – 0.12) | 0.14 (0.07 – 0.35) | 0.01 (0.002 – 0.03) | 0.19 (0.05 – 0.07) | 0.55 (0.13 – 2.12) |
| ***Total recurrent costs*** | ***9.92 (4.43 - 16.85*** | ***15.36 (8.17 - 24.57)*** | ***9.58 (3.89 - 18.77)*** | ***39.66 (8.86- 101.11)*** | ***61.44 (16.33- 147.42)*** | ***38.31 (7.79- 112.62)*** |
| ***Total costs*** | ***10.19 (4.50 - 17.90)*** | ***15.67 (8.33 - 25.37)*** | ***9.79 (3.98 - 19.52)*** | ***40.76 (9.01- 107.39)*** | ***62.66 (16.33 - 152.20)*** | ***39.18 (7.95 - 117.14)*** |
| ***Total cost, excluding GIFT device*** | ***5.19 (3.50 - 7.90)*** | ***10.67 (7.33 - 15.37)*** | ***4.79 (2.98 - 9.52)*** | ***20.76 (7.01 - 47.39)*** | ***40.66 (14.66 - 92.20)*** | ***19.18 (5.95 - 57.14)*** |

**References**

1. International Monetary Fund. Implied PPP conversion rate [Internet]. 2023 [cited 2025 Sep 24]. Available from: https://www.imf.org/external/datamapper/PPPEX@WEO/OEMDC/ADVEC/WEOWORLD
